# Gut microbiome encoded purine and amino acid pathways present prospective biomarkers for predicting metformin therapy efficacy in newly diagnosed T2D patients

**DOI:** 10.1080/19490976.2024.2361491

**Published:** 2024-06-13

**Authors:** Ilze Elbere, Zigmunds Orlovskis, Laura Ansone, Ivars Silamikelis, Lauma Jagare, Liga Birzniece, Kaspars Megnis, Kristaps Leskovskis, Annija Vaska, Maris Turks, Kristaps Klavins, Valdis Pirags, Monta Briviba, Janis Klovins

**Affiliations:** a Translational Omics Group, Latvian Biomedical Research and Study Centre, Riga, Latvia; b Faculty of Natural Sciences and Technology, Riga Technical University, Riga, Latvia; c Institute of Biomaterials and Bioengineering, Faculty of Natural Sciences and Technology, Riga Technical University, Riga, Latvia; d Baltic Biomaterials Centre of Excellence, Headquarters at Riga Technical University, Riga, Latvia; e Faculty of Medicine, University of Latvia, Riga, Latvia

**Keywords:** Gut microbiome, metformin, T2D, biomarkers, functional profile, metabolic analysis

## Abstract

Metformin is widely used for treating type 2 diabetes mellitus (T2D). However, the efficacy of metformin monotherapy is highly variable within the human population. Understanding the potential indirect or synergistic effects of metformin on gut microbiota composition and encoded functions could potentially offer new insights into predicting treatment efficacy and designing more personalized treatments in the future. We combined targeted metabolomics and metagenomic profiling of gut microbiomes in newly diagnosed T2D patients before and after metformin therapy to identify potential pre-treatment biomarkers and functional signatures for metformin efficacy and induced changes in metformin therapy responders. Our sequencing data were largely corroborated by our metabolic profiling and identified that pre-treatment enrichment of gut microbial functions encoding purine degradation and glutamate biosynthesis was associated with good therapy response. Furthermore, we identified changes in glutamine-associated amino acid (arginine, ornithine, putrescine) metabolism that characterize differences in metformin efficacy before and after the therapy. Moreover, metformin Responders’ microbiota displayed a shifted balance between bacterial lipidA synthesis and degradation as well as alterations in glutamate-dependent metabolism of N-acetyl-galactosamine and its derivatives (e.g. CMP-pseudaminate) which suggest potential modulation of bacterial cell walls and human gut barrier, thus mediating changes in microbiome composition. Together, our data suggest that glutamine and associated amino acid metabolism as well as purine degradation products may potentially condition metformin activity via its multiple effects on microbiome functional composition and therefore serve as important biomarkers for predicting metformin efficacy.

## Introduction

Metformin (dimethylbiguanide) is the first-line anti-hyperglycemic medication for the treatment of type 2 diabetes mellitus (T2D) worldwide. It has been characterized by several advantages over other types of anti-diabetic medications including low risk of hypoglycemia, decreased insulin resistance, better cardioprotection, and improved weight control, lipid profiles, and fat distribution. Apart from lowering glucose output in the liver and increasing glucose uptake in muscle, metformin effects are proven to be highly dependent on its action in the gut.^1,2^ However, more than 15–20% of patients fail to reach the glycemic target when on metformin monotherapy.^3,4^ This highlights the knowledge gap about the exact mechanisms of action of this drug and potential indirect or synergistic effects on gut microbiota and interactions with gut heterogeneity within the human population. Moreover, extended insight into the complexity of metformin mechanisms of action would aid the predictions of its efficacy and more efficient and personalized administration.

Therefore, hitherto metformin is still being studied in context with its various effects and therapeutic targets, as well as interaction with the human gut microbiome. In T2D patients, *Akkermansia muciniphila*, *Escherichia* spp., *Intestinibacter* spp., as well as others have been found to be significantly affected by metformin administration. These gut microbiome changes are resulting in increased production of short-chain fatty acids (SCFAs), regulation of bile acid metabolism, augmented integrity of the intestinal barrier, and improved glucose homeostasis of the host.^5^ Previous metabolome studies have indicated that metformin therapy is associated with tricarboxylic acid (TCA) cycle, urea cycle, glucose metabolism, lipid metabolism, or gut microbiota metabolism, however, the observed changes often are organism, patient health status, and tissue type specific.^6^

In our previous studies, we were among the first to use gut microbiome data for the prediction of possible metformin therapy efficacy.^7^ Classification of T2D patients as metformin therapy Responders and Non-responders according to changes in HbA1c levels has been done previously only in the context of other parameters, like genetic polymorphisms, urine metabolites, or DNA methylation.^8–11^ Nevertheless, the influence of compositional and functional microbiome variations on drug action, toxicity, and therapy efficacy has been highlighted and studied in the framework of pharmacomicrobiomics. Thus, the gut microbiome has emerged as an essential element for the development of precision medicine strategies.^12,13^ We found that the gut microbiome composition before starting the antidiabetic therapy could be used as a predictive tool for therapy efficacy or tolerance, however, higher sequencing depth, a larger study group, and additional analyses were needed for the nomination of more complex and precise biomarkers.^7^ The gut microbiome and microbial metabolites have been highlighted as important and prospective diagnostic and therapeutic biomarkers.^14^ Therefore, taking together the findings of our and other groups^7–11–14^ we wished to further investigate the taxonomic, functional, and metabolomic changes and differences in metformin therapy Responders and Non-responders in order to bring new knowledge for answering the three following scientific questions: (1) Can we predict the metformin therapy efficacy based on the microbial composition of Responders and Non-responders before the onset of the therapy? (2) What are the differences in metformin effects between Responders and Non-responders after 3 months of therapy? (3) What is the possible mechanism of the observed therapy efficacy within the Responder group?

## Results

### Baseline characterization of the cohort

**Figure 1.**
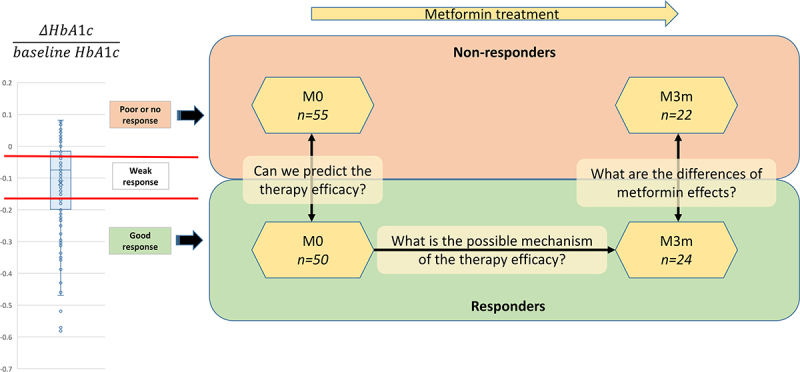
Study design and corresponding study questions. ΔHbA1c was calculated by comparing baseline values with those measured after three months of metformin treatment. M0 – before metformin therapy, M3m – after 3 months of metformin therapy.

In total, 155 newly diagnosed and metformin treatment naive T2D patients were included in this study. After calculating the A1c index for metformin therapy efficacy evaluation, we divided the participants into three similarly sized groups. The individuals with the highest or lowest therapy efficacy (i.e., Responders and Non-responders) were selected for downstream analysis to search for metformin efficacy biomarkers (Figure 1). The number of participants in both groups after 3 months of metformin therapy was lower than at baseline due to dropout or early therapy change.

The initial cohort and selected subgroups are characterized in Table 1. Despite the observed variance in prescribed metformin dose and titration algorithms, median values in all groups were equal when starting the therapy. Metformin dose at M3m time point (after 3 months of metformin therapy) is the final prescribed dose after the titration. The statistical significance of the difference in metformin dose at M3m time point could be explained by the higher dispersion of metformin dose values in the Non-responders group.

**Table 1.** Characteristics of all patients and the two subgroups selected from the extreme ends of the calculated A1c index – at baseline (M0) and after 3 months of metformin therapy (M3m).

Variable	Total (*n* = 155)	A1c index (∆HbA1c/baseline HbA1c)		p-value*
		Responders (*n* = 50)	Non-responders (*n* = 55)	
Females/males, n (%)	81/74	25/25	36/19	–
Age [years], median (IQR)	61 (15.5)	56.5 (13)	64 (16)	0.006
Baseline BMI [kg/m^2^], median (IQR)	33.8 (5.9)	33.9 (4.3)	33.6 (6.6)	0.77
BMI at M3m time point [kg/m^2^], median (IQR)	33.4 (6.4)	33.6 (6.3)	33.3 (6.5)	0.57
Baseline HbA1c [%], median (IQR)	6.9 (2.1)	9.2 (3.1)	6.3 (0.7)	9.3 × 10^−14^
Initial prescribed metformin dose [mg/day], median (IQR)	1000 (500)	1000 (237.5)	1000 (500)	0.09
Metformin dose at M3m time point [mg/day], median (IQR)	1700 (1000)	2000 (1000)	1000 (1000)	1.9 × 10^−4^

*p-value is calculated by comparing the subgroups of Responders and Non-responders. BMI – body mass index, IQR – interquartile range.

**Figure 2.**
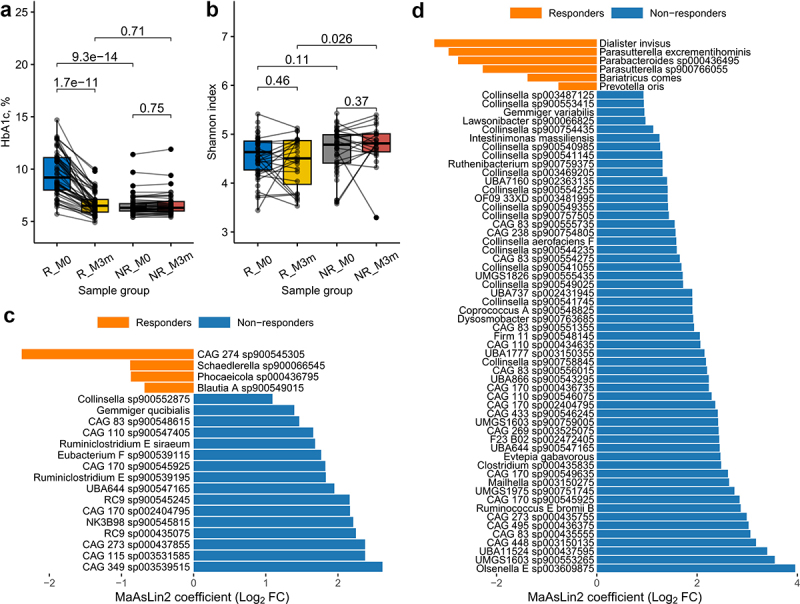
Comparison of HbA1c and the microbiome taxonomy parameters between responders and non-responders. Dynamics and differences between the analyzed groups and samples of (a) HbA1c values and (b) Shannon index. Samples from the same individuals are connected with lines. R – Responders, NR – Non-responders, M0 – before metformin therapy, M3m – after 3 months of metformin therapy. Differences in taxonomic profile at species level between responders and non-responders (c) before therapy (M0) and (d) after 3 months of metformin treatment (M3m). Barplots of Log_2_ fold change (Log_2_FC) coefficients from MaAsLin2 analysis, results adjusted for age. Negative (orange bar) and positive (blue bar) coefficient values represent species enriched in the responders and non-responders groups, respectively.

The dynamics of HbA1c and the gut microbiome sample characterization with alpha diversity are summarized in Figure 2(a,b). When comparing BMI between the groups as well as evaluating changes during metformin treatment, no significant differences were detected. Also, beta diversity did not change significantly when comparing M0 (baseline before therapy) and M3m samples within the analyzed groups.

Taking into account the highly variable nature of the gut microbiome and its interaction with host-related factors, we collected and summarized detailed data about our cohort regarding health, used medications, diet, physical activity level, and other lifestyle factors (Supplementary file 9). After evaluating the effect of all available health and lifestyle data on the gut microbiome composition of the initial patient cohort, we obtained a list of 22 potentially significant factors (Supplementary file 10), which were then compared between the analyzed groups. The only significantly different factor between Responders and Non-responders was age, which was further used as a covariate for the performed analyses of the taxonomic and functional profile of the gut microbiome. The study groups of Responders and Non-responders were balanced by all the other evaluated factors.

To validate the effects and differences observed in shotgun metagenome data, for a subgroup of patients we performed a targeted fecal metabolome analysis. Characteristics of patients included in this analysis are summarized in Table 2.

**Table 2.** Fecal metabolomics experiment study group characteristics at baseline – all patients and the two subgroups selected from the extreme ends of the calculated A1c index.

Variable	Total (*n* = 34)	A1c index (∆HbA1c/baseline HbA1c)		p-value*
		Responders (*n* = 22)	Non-responders (*n* = 12)	
Females/males, n (%)	19 (56)/15 (44)	10 (45)/12 (55)	7 (58)/5(42)	–
Age [years], median (σ)	61 (13.31)	56.5 (14.10)	65 (9.56)	0.03
Baseline BMI [kg/m2], median (σ)	33.44 (4.91)	33.44 (4.37)	32.8 (5.98)	0.93
Baseline HbA1c [%], median (σ)	7.7 (2.01)	8.5 (2.03)	6.75 (0.66)	3.69 × 10^−5^

*p-value is calculated by comparing the subgroups of Responders and Non-responders. BMI – body mass index, σ – standard deviation.

### Differences between the metformin therapy responders and non-responders

**Figure 3.**
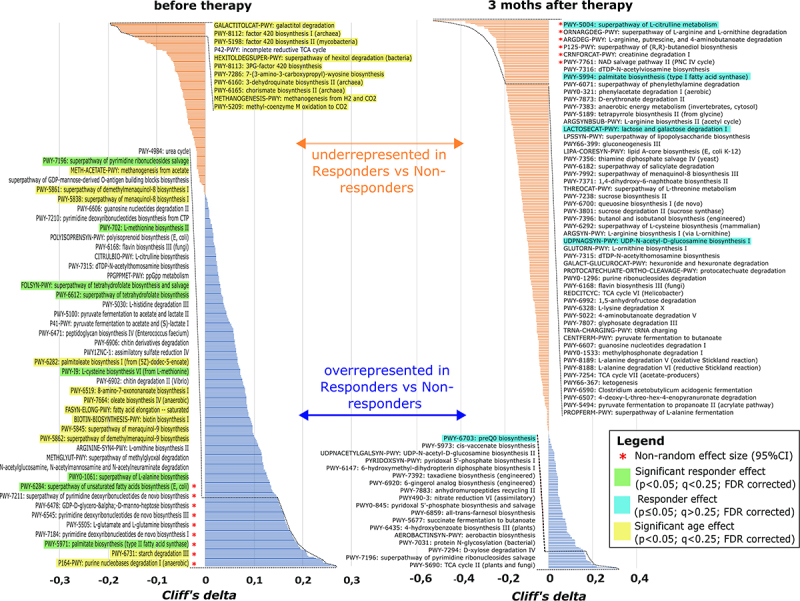
Overrepresented and underrepresented bacterial metabolic pathways in responders vs non-responders before and after metformin therapy. Non-parametric cliff’s delta values display the probability [0;1] of non-random pathway coverage differences between Responders and Non-responders. The larger the cliff’s delta deviation from 0, the more likely reads corresponding to a metabolic pathway originate from Non-responders (orange) or Responders (blue) group. Cliff delta of 0 indicates 50:50 chance of a pathway read distribution between Non-responders and Responders. The asterisk denotes statistically significant bias in pathway coverage for either Non-responders or Responders, based on 95% CI for convenience, Cliff’s delta cutoff of 0.15 and 0.2 is chosen for the written pathway names before and after the therapy, respectively. The effect size of differences in each metabolic pathway coverage is considered only for pathways that are represented by genomic reads in a full set of all annotated genes within that pathway. Color codes highlight pathways that show significantly different pathway coverage between Responders and Non-responders (green) or patient age as cofactor (yellow), when corrected for multiple comparisons using MaAsLin2 pipeline (p < 0.05; q < 0.25). Turquoise color highlights notably different pathway coverage between Responders and Non-responders 3 months after the therapy showing sign. p-value ≤0.05 but q > 0.25.

To answer our study questions, firstly, we aimed to identify any taxonomic or functional differences in the gut microbiome between the Responders and Non-responders at baseline (M0) as well as after 3 months of metformin therapy (M3m). Baseline comparison will likely highlight any prospective biomarkers for future prediction of treatment efficacy, while 3-month comparison is expected to display the main differences in compositional and functional change as a result of the therapy as well as the preexisting confounding differences in Responder and Non-responder patients before the therapy. In total, 20 differentially abundant species were detected comparing Responders vs Non-responders at baseline (Figure 2(c)), and 62 when comparing samples collected after 3-month long metformin therapy (Figure 2(d)). Similarly, when evaluating the effect size, nine significantly different bacterial metabolic pathways were detected at baseline between Responders and Non-responders, and six after 3-month long metformin therapy (Figure 3). Potential functional markers that are overrepresented in Responders compared to Non-responders before the therapy (M0) group into 1) deoxynucleotide metabolism (purine degradation and pyrimidine *de novo* synthesis); 2) carbohydrate metabolism (starch degradation & D-manno-heptose anabolism, 3) unsaturated fatty acid (palmitate) biosynthesis, as well as 4) glutamine biosynthesis. Three months after the therapy (M3m) the main differences between Responders and Non-responders are characterized by the underrepresentation of several functional pathways in Responders compared to Non-responders: 1) a multi-component pathway that involves citrulline & L-arginine conversion to L-ornithine & 4-butatenoate, 2) creatinine degradation and 3) NAD salvage. In addition to the effect size assessment, we performed a complementary analysis to identify which of the individual metabolic pathways at the M0 and M3m are consistently explained by therapy efficiency or other cofactors such as age when FDR-corrected for multiple comparisons (Supplementary file 11). While many differences between Responders and Non-responders could be attributed to patient age before the therapy (Figure 3), we confirmed that several pathways with notable effect size (pathway coverage difference) could be significantly explained by the later response to therapy. These pathways included unsaturated fatty acid (palmitate) biosynthesis as well as amino acid metabolism (Figure 3). While none of the individual pathways from metagenome data displayed significant change after FDR correction at M3m, therapy response was strongly associated with citrulline metabolism coverage, palmitate biosynthesis and lactose degradation (Figure 3).

**Figure 4.**
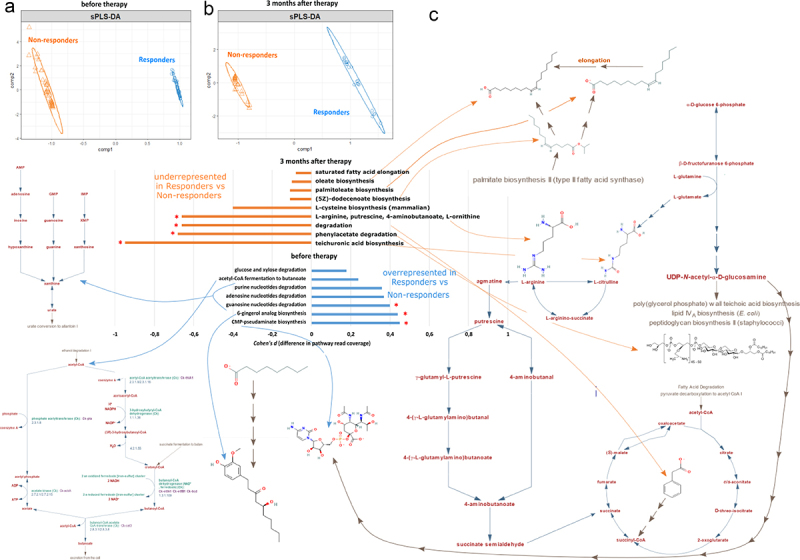
Differences in the normalized pathway read coverage between Responders and Non-responders at the baseline as well as 3 months after the therapy. Sparse partial least squares discriminant analysis (sPLS-DA) identifies components that separate Responders and Non-responders (a) before or (b) after the therapy based on microbiome functional pathway abundances using the mixOmics package in R. Further identification and visualization of the functional pathways within Responder and Non-responder defining components is depicted in panel c. Cohen values display the difference in mean pathway coverage normalized by the pooled SD. Negative Cohen values indicate decreased read coverage of a pathway in Responders (blue) vs Non-responders (orange) while positive values indicate increased coverage of a pathway in Responders vs Non-responders (blue). 95% CI denotes statistically significant effect size (differences in genomic read coverage) and is denoted by the red asterisk for the respective pathways. The effect size of differences in each metabolic pathway coverage is considered only for pathways that are represented by genomic reads in a full set of all annotated genes within that pathway. Pathways were visualized in metacyc and PubChem databases and linked to the barchart using arrows for better representation of potentially linked functions.

Since the cliff’s delta tests for changes in each of 531 annotated Humann3.0 pathways individually in an unbiased manner, such an approach may overlook individually non-significant but coordinated changes in metabolically linked sets of finer annotated pathways that may feed into each other. To search for such potentially larger combined effects of inter-linked pathway groups and functions, we employed the sPLS-DA approach to identify components that explain differences in pathway coverage between Responders and Non-responders at baseline (Figure 4(a)) as well as after 3 months (Figure 4(b)) of metformin therapy. Together, we identified seven pathways for two components at baseline that are overrepresented in Responders compared to Non-responders (Figure 4(c)). They include purine base degradation, glucose degradation as well as gingerol and pseudaminate synthesis. We further displayed the change of these pathways by calculating the difference in mean pathway coverage between three-month and baseline samples (*cohen d* metrics). We then visualized the pathways to manually assess functional linkages, e.g., shared substrates, metabolic intermediates, and products (Figure 4(c)). Interestingly, we found a cluster of purine catabolism pathways that display higher read coverage in Responders *versus* Non-responders at the baseline. Congruently, purine degradation was identified as a potential baseline functional marker in the unbiased *Cliff’s delta* test as well (Figure 3).

Furthermore, a set of nine potentially linked pathways at 3-month period were underrepresented in the Responders compared to Non-responders (Figure 4(c)). These relate to fatty acid biosynthesis and metabolic steps involved in arginine/ornithine/putrescine conversion to butanoate intermediates and to succinate (or its derivatives) which can feed into the bacterial TCA cycle. Citrulline, L-arginine, L-ornithine, and putrescine 4-butanoate metabolism were identified as potential functional markers with the highest differences in pathway coverage in pairwise comparisons between Responders and Non-responders (Figure 3). Concomitantly, they appear to be metabolically linked in our group discriminant analysis as well (Figure 4(c)). Interestingly, 3 months after the therapy, Responders displayed an underrepresentation of pathways involved in teichuronic acid synthesis compared to poor responders (Figure 4(c)) – a common component of Gram+ bacterial cell wall peptidoglycans, potentially suggesting a relative decrease in Gram+ bacterial titer or richness in therapy Responders compared to Non-responders. Moreover, UDP-N-acetyl-a-D-glucosamine is a product of glucose catabolism and feeds into lipopolysaccharide cell wall synthesis in Gram- bacteria as well as teichuronic acid synthesis of Gram+ bacteria peptidoglycans. UDP-N-acetyl-a-D-glucosamine is also a precursor for CMP-pseudaminate which is overrepresented in Responder compared to Non-responder metagenome data at the baseline (Figure 4(c)), suggesting that teichuronic acid underrepresentation in Responders’ microbiota could potentially be conditioned already at baseline and characterize future therapy efficacy.

Next, for a subset of 34 T2D patients, we performed metabolomic analysis to identify individual metabolites, their ratios or constituted pathways that differentiate Responders (*n* = 22) and Non-responders (*n* = 12) at baseline and 3 months after starting the metformin therapy. By performing linear regression analysis for time series (before treatment, after 3-month long treatment) with two-group (Responders, Non-responders) design and controlling for age, we identified 13 significant (p-value <0.05) metabolites (Table 3) in contrast Non-responders vs Responders among 53 measured molecules (Supplementary file 1). After multiple testing, eight metabolites were statistically significant (FDR <0.05), from which the greatest fold change between Responders and Non-responders are displayed by Glutamine (↑ in Responders), Ursodeoxycholic acid (↑ in Responders), Cysteine (↑ in Responders), and Isobutyric acid (↓ in Responders). Interestingly, metagenome data also highlight underrepresentation of sequences corresponding to glutamate – citrulline – arginine – butanoate pathway in Responders compared to Non-responders 3 months after the therapy (Figure 4).

**Table 3.** Statistically significant metabolites for the therapy response group (identified by linear regression model (based on *limma*) for time series data for two groups with age as a covariate).

Metabolite	logFC	P-value	FDR
Glutamine	–0.89516	0.000307	**0.015637**
Ursodeoxycholic acid	–0.75286	0.000815	**0.015637**
Isobutyric acid	0.69797	0.000902	**0.015637**
Cystine	–0.73214	0.002129	**0.027676**
Glycodeoxycholic acid	–0.57197	0.00371	**0.032531**
Propionic acid	0.57335	0.0038	**0.032531**
Acetic acid	0.53963	0.004379	**0.032531**
Tyrosine	0.67591	0.005787	**0.037614**
Butyric acid	0.51481	0.009688	0.055975
Glycolithocholic acid	–0.60614	0.024121	0.12543
Isovaleric acid	0.44986	0.029114	0.13753
Deoxycholic acid	–0.43691	0.031738	0.13753
Chenodeoxycholic acid	–0.63973	0.036626	0.1465

logFC represents log2 fold change (the change in metabolite abundance) in contrast Non-responders vs Responders, a positive value indicates upregulation, negative value indicates downregulation in Non-responders group compared to Responders group. FDR is the p-value adjusted for multiple hypothesis testing using the false discovery rate (FDR) method, FDR values < 0.05 are marked in bold.

**Figure 5.**
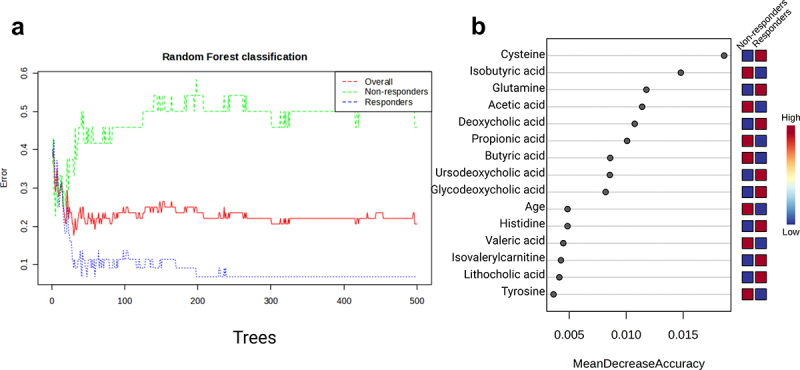
Random forest classification. (a) Random forest classification error plot, OOB error (overall – red, 0.206) is compared to the classification error of each group (responders – green (0.0682)), non-responders – blue (0.458)) with an increasing number of decision trees (n=500). (b) Variable importance plot showing the top 15 discriminatory metabolites identified by random forest technique and ranked by their contribution to classification accuracy (the mean decrease accuracy expresses how much accuracy the model losses by excluding each variable), the red color indicates the upregulation, but the blue color indicates downregulation of the metabolite in the group.

We also performed supervised Random forest (RF) machine learning analysis, including age as a possible predictor, to study the nonlinear relationships between variables and identify metabolites that could potentially predict response to metformin at baseline. Figure 5(a) shows the results of random forest classification for 500 decision trees (error plot), the OOB error is 0.206, the classification error for Response prediction is 0.0682 and for the Non-responders group, it is 0.458. This indicates that the model is suitable for predicting T2D patients who will respond well to metformin therapy in terms of efficacy, but not so suitable for predicting Non-responders. Figure 5(b) shows the Variable Importance plot, where Cysteine, followed by Isobutyric acid, Glutamine, and Acetic acid are the most influential metabolites for classification (measured by Mean Decrease Accuracy, Figure 5(b)). To note, age also was identified as a feature that contributes to response classification accuracy. Congruent with the functional metagenome analysis, glutamine (or glutamate) levels were significantly more abundant both in metabolite data (Figure 5(b)) as well as displayed overrepresentation in microbiome functional coverage at baseline in Responders compared to Non-responders (Figure 3).

**Figure 6.**
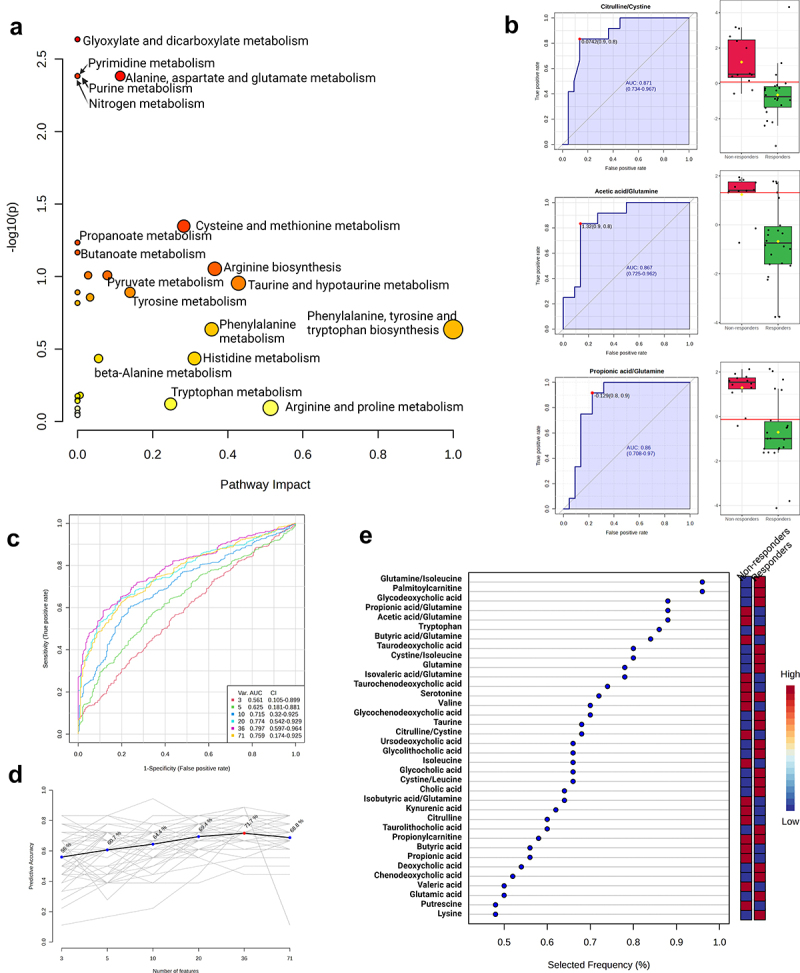
Targeted metabolomic analysis of fecal samples in metformin response groups before therapy. (a) Scatter plot representing the most relevant metabolic pathways from KEGG library arranged by adjusted p-values (obtained by Global Test pathway enrichment analysis) on Y-axis, and pathway impact values (from pathway topology analysis) on X-axis. The node color is based on its p-value and the node radius is determined based on its pathway impact values. (b) Top three biomarkers (by AUC value) from univariate ROC curve analysis, the graph on left the sensitivity (true positive rate) of the biomarker on the y-axis against its 1-specificity (false positive rate) on the x-axis, on the right graph black dots in boxplots show quantified values of biomarkers in all samples, the notch indicates 95% confidence interval around each median in metformin response groups, the mean value is showed with a yellow dot in each group, all biomarker AUC value calculations in Supplementary file 2. (c) Supervised multivariate ROC analysis curves, classification, and feature ranking method: support vector machines (SVM), the true positive rates on y-axis, the false-positive rates on x-axis. (d) Predictive accuracies (y-axis) for ROC models with different counts of features (x-axis), the model using 35 features reached the highest Predictive Accuracy (red dot). (e) Top 35 features from the most accurate ROC model (predictive accuracy 70.7%), variant importance plot based on feature Selected Frequency (%), it refers to the percentage of times a feature (metabolite) was selected as important or influential by the support vector machine (SVM) classifier. The red color on the right panel for response indicates the upregulation, but the blue color indicates the downregulation of the feature in the group. All biomarker importance calculations from this model are in Supplementary file 3.

Next, we performed Pathway analysis (Figure 6(a), Supplementary file 2), univariate (Supplementary file 3A) and multivariate (Supplementary file 3B) Biomarker analysis (Figure 6(b–e)) for targeted metabolites measured in fecal samples collected before metformin therapy (M0). Most significant differences in pathways between response groups were displayed in Glyoxylate and dicarboxylate metabolism, Nitrogen metabolism, Purine and Pyrimidine metabolism (Figure 6(a)) which were also significant metagenome functional biomarkers for therapy efficacy (Figures 3 and 4) and could suggest that baseline microbial deoxynucleotide abundances may be a good predictor for metformin therapy efficacy. In addition, Alanine, aspartate, and glutamate pathway displays pronounced differences between Responders and Non-responders at baseline (Figure 6(a)) as well as changed metabolite ratios (e.g., acetic acid/glutamine, propionic acid/glutamine) (Figure 6(e)). Importantly, our non-targeted metagenome analysis for microbiome functional coverage identified glutamine biosynthesis being overrepresented in Responders vs Non-responders at baseline (Figure 3) and implies downstream functional links with arginine/citrulline/putrescine metabolism which signifies differences between Responders and Non-responders 3 months after metformin therapy (Figure 4(c)). Taken together, purine and glutamate pathways could be used as a potential predictor for metformin efficacy and confound subsequent metformin effects during the course of the therapy in each response group.

**Figure 7.**
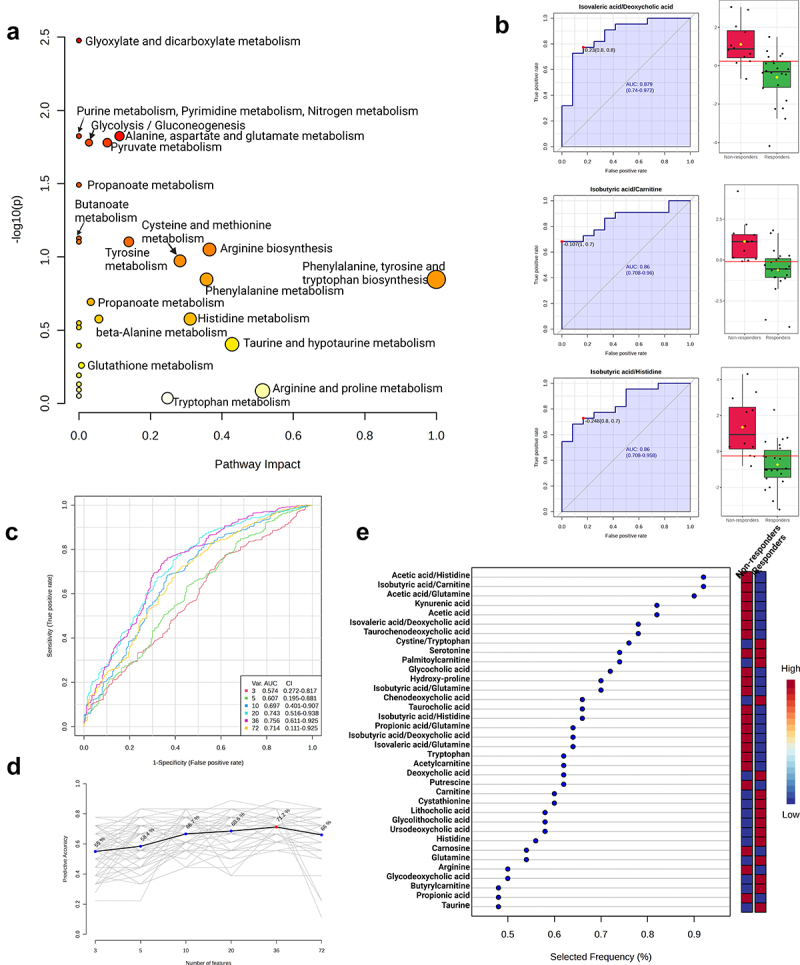
Targeted metabolomic analysis of fecal samples in metformin response groups after 3 month long therapy. (a) Scatter plot representing the most relevant metabolic pathways from KEGG library arranged by adjusted p-values (obtained by global test pathway enrichment analysis) on Y-axis, and pathway impact values (from pathway topology analysis) on X-axis. The node color is based on its p-value and the node radius is determined based on its pathway impact values. (b) Top three biomarkers (by AUC value) from univariate ROC curve analysis, the graph on left the sensitivity (true positive rate) of the biomarker on the y-axis against its 1-specificity (false positive rate) on the x-axis, on the right graph black dots in boxplots show quantified values of biomarkers in all samples, the notch indicates 95% confidence interval around each median in metformin response groups, the mean value is showed with a yellow dot in each group, all biomarker AUC value calculations in Supplementary file 4. (c) Supervised multivariate ROC analysis curves, classification, and feature ranking method: support vector machines (SVM), the true positive rates on y-axis, the false-positive rates on x-axis. (d) Predictive accuracies (y-axis) for ROC models with different counts of features (x-axis), the model using 36 features reached the highest predictive accuracy (red dot). (e) Top 36 features from the most accurate ROC model (predictive accuracy 68.3%), variant importance plot based on feature selected frequency (%), it refers to the percentage of times a feature (metabolite) was selected as important or influential by the support vector machine (SVM) classifier. The red color on the right panel for response indicates the upregulation, but the blue color indicates the downregulation of the feature in the group. All biomarker importance calculations from this model are in Supplementary file 5.

Since glutamate can be converted to citrulline and thus affect downstream butanal and butanoate production (Figure 4c), we aimed to further evaluate the differences between Responders and Non-responders 3 months after metformin treatment. To this end, we used our metabolome data to perform the Pathway analysis (Figure 7(a), Supplementary file 4) as well as univariate (Supplementary file 5A) and multivariate (Supplementary file 5B) Biomarker analysis (Figure 7(b–e)) for fecal samples collected at the second time point (M3m). The most significant changes were observed in glyoxylate and dicarboxylate metabolism (Figure 7(a)) which involve TCA cycle intermediates such as succinate, malate, fumarate, glyoxylate, etc. Interestingly, glutamate conversion to citrulline, arginine, putrescine, and further to aminobutanoate can feed into the TCA cycle as predicted from our metagenome analysis (Figure 4(c)). Thus, the observed changes in microbiome functional coverage (Figure 3) may substantiate both the differences in metabolic pathways (Figure 7(a)) as well as metabolite ratios (Figure 7(e)) between Responders and Non-responders after 3-month long metformin therapy.

### Metformin-induced changes specific to the responders group

**Figure 8.**
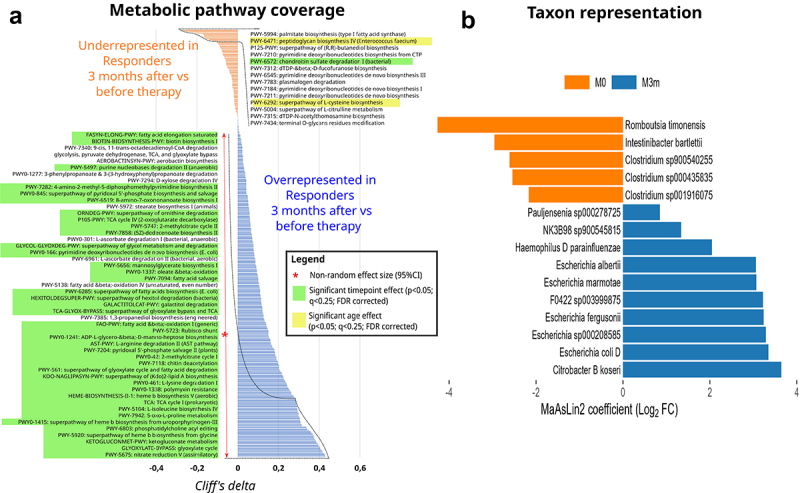
Changes in the representation of taxonomic and metabolic pathway profile in the Responders group during the metformin treatment. (a) Non-parametric cliff’s delta values display the probability [0;1] of non-random pathway coverage changes 3 months after metformin therapy compared to the baseline before therapy. The larger the cliff’s delta deviation from 0, the more likely the reads corresponding to a metabolic pathway are detected before the therapy (orange) or after (blue) the therapy. Cliff delta of 0 indicates equal chance of a pathway reads to be detected before as well as after the therapy. The asterisk denotes statistically significant effect size in metabolic pathway read coverage based on 95% CI. For convenience, Cliff’s delta cutoff of 0.3 is chosen for displaying written pathway names. Color codes highlight pathways that show significantly different pathway coverage between baseline and 3 month timepoint (green) or patient age as cofactor (yellow), when corrected for multiple comparisons using MaAsLin2 pipeline (p<0.05; q<0.25). (b) Bacterial taxa abundance displayed in barplots of Log2 fold change (Log2FC) coefficients from MaAsLin2 analysis, results adjusted for age. Negative (orange bar) coefficient values represent taxa more abundant before therapy (M0), whereas positive (blue bar) - after 3-month therapy (M3m).

Finally, we analyzed metformin effects specific to the Responders group with the aim to detect possible mechanisms of the augmented therapy efficacy in this patient subgroup. According to metagenome data, Responders display significant overrepresentation of several functional pathways 3 months after starting the metformin therapy compared to baseline: 1) nitrate reduction, 2) TCA cycle and its intermediates glyoxylate, ketogluconate, 3) heme biosynthesis, 4) phosphatidylcholines which are part of bacterial membranes, and 5) synthesis of amino acids proline and isoleucine (Figure 8(a)). Crucially, most of the pathways with the greatest change in genomic coverage (effect size) were significantly different between the time points rather than Responder age-dependent even after the FDR correction. We, furthermore, observed metformin-induced changes in 15 bacterial species (Figure 8(b)) with Gram- bacteria (*Citrobacter* and various *Escherichia* species) increasing but several Gram+ Clostridiaceae species (*Intestibacter, Clostridium, Romboutsia)* decreasing after the therapy.

**Figure 9.**
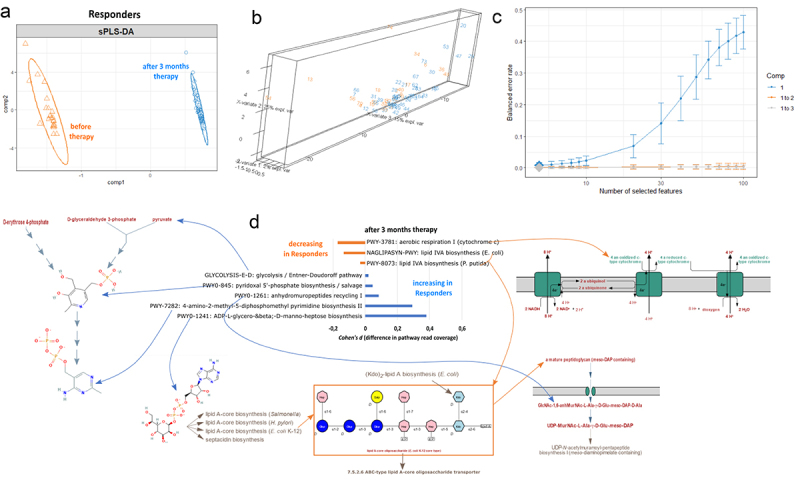
Increase or decrease in the normalized pathway read coverage between baseline and 3 month cohorts in responders. (a) Sparse partial least squares discriminant analysis (sPLS-DA) identifies components that separate responders before and after the therapy based on microbiome functional pathway abundances using the mixOmics package. (b) 3D visualization identifies a single component that separates responders before and after the therapy. (c) 8 microbiome genomic pathways (selected features) within component 1 demonstrate the lowest balanced error rate in discriminating differences between baseline and 3 month responses. Visualization of other components (b) and varying feature number within each component (c) does not improve discrimination of responders at baseline and after the therapy. Further identification and visualization of the functional pathways within component 1 characterize the therapy effect in responders and are depicted in panel d. Cohen values display the difference in mean pathway coverage normalized by the pooled SD. Negative Cohen values indicate decreased read coverage of a pathway 3 months after the therapy (orange) while positive values indicate increased coverage after the treatment (blue). 95% CI denotes statistically significant effect size (differences in genomic read coverage) and is denoted by the red asterisk for the respective pathways. The effect size of differences in each metabolic pathway coverage is considered only for pathways that are represented by genomic reads in a full set of all annotated genes within that pathway. Pathways were visualized in metacyc and PubChem databases and linked to the barchart using arrows for better representation of potentially linked functions.

Next, in order to capture prospectively linked or coordinated changes in metabolic pathways, we employed the sPLS-DA approach to identify components that explain changes in microbial functional pathway genomic coverage between baseline and the 3-month time point within the Responder group (Figure 9(a)). We identified two components that explain 25% and 2% of the variation in metabolic functions over 3 months of therapy (Figure 9(b)) and together contain eight bacterial pathways determined by the lowest error rate in our PSL model (Figure 9(c)). Interestingly, we identified an overrepresentation of functions contributing to pyrimidine synthesis as well as pathways leading to D-manno-heptose synthesis which is a precursor for bacterial lipid-A core lipopolysaccharide synthesis in Gram- bacteria (Figure 9(d)). Surprisingly, our analysis also demonstrated underrepresentation of bacterial lipidA synthesis while anhydromuropeptide recycling pathway displayed increased read coverage in Responders (Figure 9(d)). Since anhydromuropeptides are degradation products of lipidA (Figure 9(d)), data suggest shifts in the potential balance between lipidA synthesis and degradation. Additionally, we also identified metformin-induced changes in phosphatidyl acyl editing (Figure 8(a)). Therefore, we hypothesize that the observed collective changes in bacterial membrane/cell wall component biosynthesis and degradation may be linked with taxon-specific shifts in microbiome composition such as the observed increase in Gram- taxa in metformin Responders (Figure 8(b)). However, this hypothesis requires further studies and characterization.

**Figure 10.**
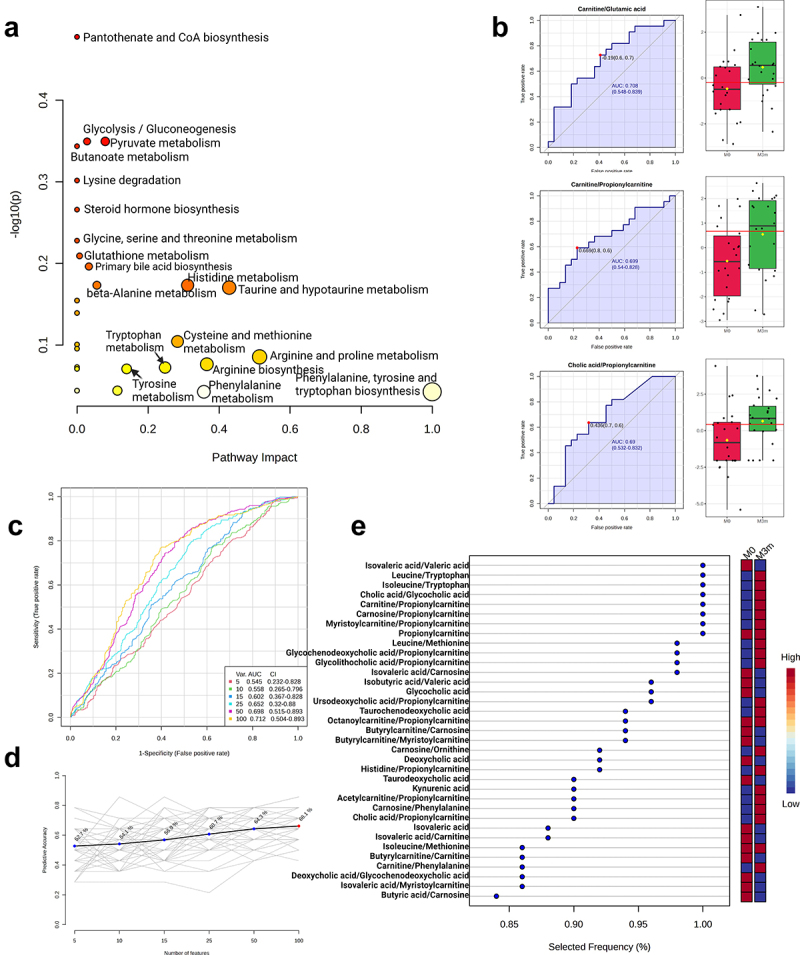
Targeted metabolomic analysis of fecal samples in responders before and after 3-month-long metformin treatment. (a) Scatter plot representing the most relevant metabolic pathways from KEGG library arranged by adjusted p-values (obtained by global test pathway enrichment analysis) on Y-axis, and pathway impact values (from pathway topology analysis) on X-axis. The node color is based on its p-value and the node radius is determined based on its pathway impact values. (b) Top three biomarkers (by AUC value) from univariate ROC curve analysis, the graph on left the sensitivity (true positive rate) of the biomarker on the y-axis against its 1-specificity (false positive rate) on the x-axis, on the right graph black dots in boxplots show quantified values of biomarkers in all samples, the notch indicates 95% confidence interval around each median in metformin response groups, the mean value is showed with a yellow dot in each group, all biomarker AUC value calculations in Supplementary file 6. (c) Supervised multivariate ROC analysis curves, classification, and feature ranking method: support vector machines (SVM), the true positive rates on y-axis, the false-positive rates on x-axis. (d) Predictive accuracies (y-axis) for ROC models with different counts of features (x-axis), the model using 36 features reached the highest predictive accuracy (red dot). (e) Top 36 features from the most accurate ROC model (predictive accuracy 63.4%), variant importance plot based on feature selected frequency (%), it refers to the percentage of times a feature (metabolite) was selected as important or influential by the support vector machine (SVM) classifier. The red color on the right panel for response indicates the upregulation, but the blue color indicates the downregulation of the feature in the group. All biomarker importance calculations from this model are in Supplementary file 7.

In order to compare the changes in microbiome functional pathways with metabolite data, next we performed the Pathway analysis (Figure 10(a), Supplementary file 6), univariate (Supplementary file 7A) and multivariate (Supplementary file 7B) Biomarker analysis (Figure 10(b–e)) contrasting M3m and M0 samples specifically in the Responders group. Metabolite pathway analysis displayed significant changes in the cluster annotated as porphyrin and chlorophyll metabolism (Figure 10(a)). Given that the iron-containing porphyrins are called hemes (while chlorophylls are Mg-containing porphyrins), the metabolic data substantiate functional changes in the genome coverage. Responders demonstrate decrease in read coverage related to aerobic respiration functions (Figure 9(d)) which may be linked to the overrepresentation in heme biosynthesis which contribute the component to electron transport chain during aerobic respiration (Figure 8(a)). Furthermore, Responders demonstrate notable changes in glutamine, glutamate, aspartate and alanine metabolism (Figure 10(a)) which are consistent with the observed differences between Responders and Non-responders 3 months after the therapy (Figure 4(c)). Moreover, while these compounds can be converted to butanoate/butanoic acid derivatives and feed into the TCA cycle (Figure 4(c)), we also observed 3-methylbutanoic (syn. isovaleric) acid ratios as one of the most strongest predictors of therapy effects within the Responder group (Figure 10(e))

## Discussion

Search for diagnostic and prognostic biomarkers is a significant and ongoing research direction in the context of T2D and metformin therapy efficacy and tolerance. From the scope of pharmacomicrobiomics and precision medicine, the gut microbiome and its elements offer the possibility to detect highly informative and noninvasive biomarkers that can also be combined with clinical data for increased accuracy as well as used for further development of microbiome modification approaches.^12–14^ Moreover, insights into microbiome changes during the therapy may highlight potentially novel mechanisms of metformin action by modulating gut microbiome and functions that are important in therapy outcome. In this study, we performed shotgun metagenomic and targeted metabolomics analyses of the gut microbiome in newly diagnosed and treatment-naive T2D patients before starting metformin treatment and after 3 months of therapy, further focusing on subgroups of Responders and Non-responders defined by changes in HbA1c during a time period of 3 months and selected from extreme values of A1c index distribution. Our aim was to address three study questions as defined in the introduction and outlined in Figure 1.

### Can we predict the metformin therapy efficacy based on the microbial composition of responders and non-responders before the onset of the therapy?

Our cohort presents a significant advantage and opportunity to test samples collected before the start of the antidiabetic therapy and evaluate microbial signatures that could be used as biomarkers to predict the therapeutic efficacy or could be selected as targets for future microbiome modulation approaches.

The gut microbiome of Responders at baseline was enriched with four different species, including one from genus *Blautia*, which has been already associated with better metabolic health and mostly has been described to be reduced in T2D or obese patients when compared to controls. The abundance of this genus has also been reduced in patients with colorectal cancer, but increased in IBD, suggesting species and strain-level effects. Nevertheless, the results from numerous studies suggest various probiotic characteristics of *Blautia* spp.^15^ Interestingly, despite the higher baseline HbA1c in the Responders group in our study, a negative correlation between the abundance of *Blautia* and the levels of fasting plasma glucose and HBA1c has been reported.^16^ Moreover, in previous studies with antidiabetic medication, metformin therapy increased the abundance of this SCFA producing genus.^5^ Results from research on *B. wexlerae* in mouse model have indicated a possible antidiabetic properties of this genus as ability to produce several metabolites that can modulate the remaining gut microbiome and are beneficial for the host, such as acetylcholine, L-ornithine, and S-adenosylmethionine. Interestingly, our metagenome analysis demonstrated that the pathway encoding L-ornithine biosynthesis from L-glutamate was also enriched in Responders compared to Non-responders before therapy (Figure 3). Metabolites produced by *Blautia* species such as *B. wexlerae* are implicated in the increase of other bacteria, for example, *Akkermansia muciniphila*,^17^ which in turn is involved in the metabolism of carbohydrates into short-chain fatty acids,^18^ and is one of the most pronounced signatures of metformin therapy.^19,20^ Unsaturated fatty acids and palmitate were also amongst the most enriched functional pathways in Responders vs Non-responders before the therapy (Figure 3). Thus, inter-specific microbial interactions may potentially manifest in cascading modulations on metabolite composition with hardly discernible cause and effect due to metabolic feedback on microbiota components.

We also observed numerous pathways related with purine degradation to ureate to be overrepresented in Responders before the therapy (Figure 4(c)). Additionally, other studies report several intermediates of purine breakdown (xanthosine, inosine, urate) to be significantly elevated in T2DM mice with metformin treatment.^21^ Congruently, purine metabolism was also significantly different in our metabolite profiling between Responders and Non-responders (Figure 6), potentially indicating that these changes in host metabolism could be explained by differences in microbiome functional composition. Furthermore, purine catabolites could potentially be good predictive markers for therapy efficacy and purine metabolism may contribute to the mechanism of action of metformin.

Microbial pathway coverage of L-glutamine and L-glutamate biosynthesis was significantly enriched in Responders before therapy compared to Non-responders (Figure 3). Moreover, we also measured increased glutamine levels and ratios for glutamine relative to other metabolites (Figure 6), suggesting a putative role of microbiome composition in the host metabolism of glutamine and glutamate in our study. Interestingly, dietary supplementation with glutamine has been demonstrated to increase IgA+ plasma cells and sIgA levels in the ileum of mice.^22^ Our previous studies in human subjects have indicated that metformin antidiabetic effects and modulation of gut microbiota could be mediated through sIgA-related mechanisms,^23^ and it has been demonstrated that metformin therapy increases the population of IgA-producing immune cells in mice.^24^ The differences and changes in glutamine metabolism present a possible element explaining the microbiome mediated mechanism for the observed changes in sIgA levels during metformin therapy. Glutamine also regulates gut intestinal barriers by being N-acetyl-glucosamine and N-acetyl-galactosamine donor for mucin biosynthesis,^25^ suggesting a complementary mechanism to *A.muciniphila*’s beneficial impact on the intestinal mucus layer associated with metformin treatment. Interestingly, we observed that Responders display enrichment for CMP-pseudaminate which is synthesized from N-acetyl-glucosamine (Figure 4) and used for some bacteria like *H. pilori* to decorate its flagellin to modulate cell-to-cell interactions.^26^ Together, our data suggest glutamine as an important biomarker for metformin efficacy and, based on other studies, may condition metformin activity via its multiple effects on microbiome composition and functions.

When comparing the results of this study with our previous pilot study for evaluating the differences in the taxonomic profile of the gut microbiome between metformin therapy response groups at baseline, we can see some of the results overlapping. For example, the gut microbiome of the Responders group (both at baseline and after 3 months of therapy) is enriched with species from the *Lachnospiraceae* family, and with species from the *Eubacterium* genus in the Non-responders group.^7^ However, despite the similarities in higher taxonomic levels, data at the species level are more diverse. The incongruity between these studies could be explained by differences in sample sizes, used statistical methods, and, most importantly, reference databases for microbiome classification.

### What are the differences in metformin effects between responders and non-responders after three months of therapy?

We found significant differences in metabolites within glutamate, glutamine, alanine, and aspartate pathways (Figure 7) which is congruent with the observed underrepresentation in read coverage of other amino acids and their derivatives (proline, citrulline, arginine, ornithine) (Figure 3) in the Responders group, highlighting the link between gut microbe functional composition and resulting metabolite profile. Given the central role of glutamate in regulating the flux of other amino acids by gut microbes,^27^ these data suggest that the observed differences in metabolites could be, at least in part, caused by metformin effects on microbial composition rather than host alone. Interestingly, a decrease in phenylalanine, tyrosine, citrulline, arginine as well as other amino acid abundances was detected after metformin therapy in other studies as well.^28^ In addition to the differences in amino acid profile and the urea cycle metabolites, we also identified changes in the ratios of acetic, isobutyric and isovaleric (3-methylbutanoic) acids relative to other metabolites (Figure 7). This is consistent with the observed increase in butyrate, acetate, and valerate during metformin therapy as shown in the study by Mueller *et al*.^29^ Furthermore, one of the possible biomarkers for the Non-responders group could be the increased presence of species from the *Collinsella* genus. Although at baseline only one species was more abundant in the Non-responders group than in the Responders, after 3 months of metformin treatment 15 different *Collinsella* species were represented in higher abundance specifically in the Non-responders group. These changes and differences in the taxonomic profile can be linked with the functional and metabolic profile, as our data present increased genomic presence of superpathway of L-citrulline metabolism in the Non-responders group, and *Collinsella* genus have been previously associated with higher microbiome gene abundance related to the arginine-deiminase activity and citrullination processes.^30^ *Collinsella* spp. has also been associated with increased levels of insulin, triglycerides, low-density lipoproteins, and other metabolic markers in various study groups,^31–33^ which could suggest the possible mechanisms for further exploration behind the reduced therapy efficacy. This difference in the abundance of the *Collinsella* genus could also be a marker validating therapy efficacy in the Responders group, as increased abundance of it has been characteristic of T2D patients when compared with healthy controls.^34^

Metagenome data suggest that saturated fatty acid (palmitate) synthesis is overrepresented in Responders compared to Non-responders before the therapy but the reverse is true 3 months after the therapy (Figure 3), suggesting that metformin has a differential effect on bacterial genomic representation of unsaturated fatty acid biosynthesis which is notably decreasing in Responders (corroborated in Figure 8) compared to Non-responders during the 3-month treatment. Palmitate and other saturated free fatty acids have been previously associated with impaired insulin secretion, impaired insulin sensitivity, and glucose intolerance^35^ as well as levels of palmitate are positively correlated with levels of HbA1c in T2D patients.^36^ Therefore, our results could be explained by the higher initial HbA1c levels within the Responders cohort. Moreover, metformin has demonstrated protective effects against various palmitate-related harmful processes.^37,38^ The observed reduction of palmitate biosynthesis could indicate another gut microbiome-related mechanism for the beneficial effects of the metformin treatment. Although we did not measure the levels of palmitate directly, the observations within the metagenome data were partially validated as we observed differences in levels of palmitoylcarnitine (Figure 6(e)).

While the differences in microbial functional pathway genomic coverage between Responders and Non-responders before the therapy do not mirror the differences between the same groups 3 months after the therapy, clustering of the metabolic profiles hint at metformin-independent but responder group-specific differences in purine as well as phenylalanine, tyrosine, tryptophan metabolic pathways (contrast Figures 6 and 7). Tryptophan is known to be an important regulator of intestinal barriers and widely metabolized by gut microbes to produce indole, serotonin kynurenine (reviewed in^6^). In support of this, the abundances of tryptophan derivatives and their ratios were strong predictors of the overall differences between Responders and Non-responders 3 months after the therapy (Figure 7). However, the metagenome analysis does not highlight phenylalanine, tyrosine, or tryptophan pathways to be different between Responders and Non-responders before or after the therapy (Figure 3). Together, this suggests that the observed differences in phenylalanine, tyrosine, and tryptophan metabolism are likely to originate neither from direct effects of metformin on the host nor metformin effects on microbial functional composition but instead indirect interactions between yet unidentified host factors and microbiome before the therapy.

### What is the possible mechanism of the observed therapy efficacy within the responder group?

While metformin effects on human metabolites are tissue-type specific,^21^ here we hoped to characterize the potential functional changes within the gut microbiome in response to metformin and capture the changes in metabolite profile that could illuminate microbiome-dependent metformin mechanisms of action. By studying yeast and human cell lines, Li *et al*.^39^ suggested that metformin acts through stabilizing appropriate redox states in heme and other porphyrin-containing groups to control cellular metabolism. In agreement with this study, we found that microbial heme b biosynthetic pathway displays increased coverage in Responders after the 3-month therapy (Figure 8(a)) but is accompanied by a decrease in NADH- and *cytochrome c-* dependent aerobic respiration (Figure 9). Pantothenate and CoA biosynthesis appears to be among the most significant changes in metabolite functional clusters in our metabolomics dataset (Figure 10), which coincides with the results from metagenomics, as pantothenate (vitamin B5) and CoA are important elements in heme biosynthesis.^40^ Moreover, metformin administration in the Responders group increased the abundance of five species from the *Escherichia* genus, which coincides with the general effects of metformin therapy reported previously.^29^ *In vitro* studies have demonstrated that metformin effects on the observed increase of *Escherichia* spp. are most likely indirect,^20^ however, metformin can alter the chemotaxis and flagellar motility of *E.coli*.^41^ Nevertheless, *E.coli* has been shown to be a porphyrin donor, which further can enhance the growth of beneficial bacteria, such as *Bacteroides* spp.^42^ Porphyrins are also required for heme biosynthesis, suggesting that the observed changes in microbial functional pathway coverage may be further explained by the characteristics of certain taxa.

Butanoate metabolism appears to be significantly affected by metformin during 3-month therapy: butanoic acid is increasing while isobutanoate and isovaleric acid are both decreasing in Responders (Figure 10). Given the role of glutamate in butanoate metabolism, the previously characterized differences in glutamate levels (Figure 7) and the genomic coverage of corresponding microbial pathways (Figures 3 and 4) may potentially explain the differences in the butanoate pathway. Moreover, these results support the reported metformin effects on butanoate^29^ and suggest the involvement of TCA cycle in butanoate synthesis (Figure 8).

While pyridoxal and pyrimidine synthesis appears to be significantly increased in Responders (Figures 8(a) and 9), these compounds were not analyzed in the metabolome of Responders. Conversely, we found changes in phenylalanine, tyrosine, and tryptophan metabolic pathways (Figure 10) but did not identify strong and significant changes in microbial genomic functional coverage (Figures 8(a) and 9) for these exact pathways, suggesting that these could be indirect host metabolic changes rather than directly linked to microbiota genomic functions. Other pathways linked to amino acid metabolism displayed more congruent changes in microbiome genomic coverage as well as fecal metabolite analysis. For example, arginine and proline pathways as well as lysine degradation were significantly altered in both metabolome (Figure 10(a)) and microbial metagenome analysis (Figure 8(a)), suggesting that these changes in metabolome could potentially be explained by functional shifts in microbiome.

### General discussion

The main limitation of this study is that only a subset of samples has data about the fecal metabolite profile; however, the strong consensus with shotgun metagenomics data increases the validity of our results. Another limitation is the reduced number of samples available after 3 months of metformin therapy (M3m); therefore, additional studies should be performed to more precisely evaluate the long-term treatment-induced or response-related differences in the gut microbiome and other health-related markers between therapy Responders and Non-responders.

Unfortunately, we did not control for the use of probiotics, prebiotics, or fermented foods during the study; however, we collected information on other health and lifestyle data, such as regularly used medications, use of antibiotics before the study, general diet, physical activity level, and others. We employed the available information to evaluate the possible effects of the specific factors on the gut microbiome in general, as well as compared them between the analyzed study groups. Due to the highly variable nature of the gut microbiome and its interaction with host-related factors, we did not perform matching of cofactors between the Responders and Non-responders as that would result in up to 75% reduction in study groups significantly reducing the power of the study. While matching of study groups is generally recommended in microbiome studies,^43^ there are significant concerns raised about the applicability of this approach in observational studies with a risk of introducing novel biases.^44^ It has been demonstrated that a regression model, with confounding variables, is more powerful in detecting treatment effects than a matched study design.^45^ It is significant to note that we did not exclude participants who reported a recent history of antibiotic treatment, due to two main reasons: (1) these data were self-reported and were not available for all participants, and (2) it has been reported that T2D patients have a higher prevalence of antibiotic therapy history than general population even up to 15 years before the diagnosis of T2D.^46^ Thus, we would exclude a significant confounding factor that shapes the gut microbial content of T2D patients. Therefore, with the aim to report data applicable to real-world clinical settings and not to introduce novel biases, these participants were included in the analyses.

In addition to the primary results of this study, our analysis of possibly confounding factors highlighted age as a significant element differentiating between the therapy Responders and Non-responders. The lower age in the Responders group within this study corresponds to our observations in T2D patients previously.^7^ Interestingly, other studies evaluating the therapeutic efficacy of metformin have concluded that older age is a predictor for better treatment response along with other factors, such as baseline HbA1c levels, BMI, and disease duration.^47,48^ These results are opposite to our data, and this could be explained by some population-specific or gut microbiome-mediated effects which need to be investigated further. Nevertheless, based on data from our and other studies, age is a significant factor for the therapeutic response of metformin and should be taken into account when developing precision medicine algorithms, biomarker sets, or even microbiome modulation approaches.

## Conclusions

The data on fecal metabolite profile were congruent and supported the results of microbiome shotgun metagenomics and their encoded functions. This strongly suggests that the subgroup of samples used for targeted metabolomics is likely to be representative of the entire longitudinal cohorts of Responders and Non-responders. We identified purine degradation, glutamate metabolism, and unsaturated fatty acids as potential predictors (biomarkers) for prospective metformin efficacy. These pathways may condition the changes in microbiome composition and functions, resulting in modulation of porphyrin and butanoate pathways, lipidA synthesis as well as the metabolism of phenylalanine, tyrosine, and tryptophan in Responders.

## Patients and methods

### Study design

The study involved treatment-naive newly diagnosed T2D patients recruited in collaboration with the Genome Database of Latvian population (LGDB)^49^ in the framework of the longitudinal OPTIMED cohort. The full list of inclusion and exclusion criteria for this cohort is summarized in Supplementary file 8. Informed consent was obtained from all participants at the beginning of the study. T2D patients were treated with metformin monotherapy as prescribed by an endocrinologist (individual dosage, titration, etc.). The study was carried out in accordance with the principles of the Declaration of Helsinki, and approved by the Central Medical Ethics Committee (1/19-10-22).

Stool samples were collected in two aliquots by participants at home (at predetermined time points), using sterile collection tubes without buffer, and within 24 hours delivered to the closest clinical or research laboratory where samples were frozen at –80°C. Samples used in this study were coded as follows: M0 – before metformin treatment; M3m – after 3 months of metformin therapy.

Associated information about each patient included health and therapy-related data (registered by their endocrinologist), self-reported information about lifestyle, health, and diet, and biochemical analysis (performed before therapy (baseline) and after 3 months). Therapy efficacy was assessed by calculating the “A1c index” (∆HbA1c/baseline HbA1c), which has been used in a number of previous studies^50,51^ and takes into account the diverse baseline HbA1c values. The participants were divided into three groups of similar size according to the A1c index: (1) Responders (R): -0.29 ± 0.11, (2) Intermediate/Weak responders (WR): -0.11 ± 0.14, (3) Non-responders (NR): 0.007 ± 0.03. Groups representing both extremes of the therapy response (Responders and Non-responders) were used for further comparisons and detailed analyses.

### Microbial DNA processing

Microbial DNA from the stool samples was extracted using the MagPure Stool DNA LQ Kit (MGITech) reagent kit and the automated platform MGISP-960 (MGI Tech). Further shotgun metagenomic library preparation was done by fragmenting the DNA at 400 bp (Covaris) and following the manual of the MGIEasy Universal DNA Library Prep Set (MGI Tech Co. Ltd). The library preparation included the following sample processing steps: (1) end repair and A-tailing after the physical fragmentation, (2) Barcode Adapter ligation and clean-up with MGIEasy DNA Clean Beads, (3) amplification and clean‑up. Negative controls for DNA extraction, library preparation, and sequencing were included in sample analysis batches as part of laboratory standard operating procedures for shotgun metagenomics. After that, the appropriate count of libraries (to obtain the planned read count) was pooled and each pool was normalized to 330 ng in a volume of 48 μl. The pooled libraries were further annealed and circularized with splint oligo. Further, DNA nanoballs were created with rolling circle amplification. The end-products were sequenced using DNBSEQ-G400RS sequencing platform (~30 M reads/sample).

### Shotgun metagenome data processing

Raw data from the sequencer were processed as follows: Adapter clipping and read trimming were performed with fastp 0.20.0. Reads shorter than 100 bp were removed. Host read removal was performed by aligning reads with bowtie2 (version 2.3.5.1) against GRCh38 (ensembl release 108) reference genome. The functionality from the remaining sequences of gut microbiome samples was analyzed using the HUMAnN3 pipeline.^52^ For the taxonomic analysis reads 100 bp or longer were classified with kraken (v2.1.2)^53^ against the Unified Human Gastrointestinal Protein (UHGP) catalog^54^ and setting kraken’s confidence threshold to 0.1. Read abundance reestimation on the species level was performed with bracken (v2.7)^55^ by setting the read number threshold to a value of 10.

### Analysis of the taxonomic profile, anthropometric parameters, and other patient-related factors

Alpha diversity was evaluated by calculating the Shannon index. Beta diversity was analyzed with the Permutational Multivariate Analysis of Variance (PERMANOVA) using distance matrices based on the Bray-Curtis dissimilarity index as implemented in R (package “vegan”). Canonical correspondence analysis (CCA) was implemented to evaluate the effect of lifestyle and health-related factors on the gut microbiome within the analyzed cohort. Further, to ensure that the study groups of interest are balanced, the significant variables from the CCA were tested for significant differences when comparing the groups of Responders and Non-responders. Shannon index, HbA1c values, and variables from CCA between sample groups were compared using the T-test, Wilcoxon test, or chi-squared test as appropriate, paired tests used when appropriate. Comparisons and visualization were performed using R (v.4.2.3). Analyses for differential abundance were performed using the Microbiome Multivariable Associations with Linear Models (MaAsLin2) tool, adjusting for age and for the patient ID as random effect in longitudinal contrasts.^56^ Only taxa present in ≥10% of the analyzed samples in abundance ≥0.01% were used for MaAsLin2. Correspondingly to other microbiome studies and the most recent recommendations by the MaAsLin2 developers, the q-value threshold was set at 0.25 and p-value threshold at 0.05.^56–58^

### Functional analysis of metabolic pathways from shotgun metagenome data

The HUMAnN3 *pathabundance* output file contains normalized pathway coverage for each sample that is proportional to the number of complete “copies” of the entire pathway in each sample. Taxon-independent combined coverage for each of 531 annotated pathways was used to contrast Responders and Non-responders before metformin therapy (baseline) and 3 months after the onset of the therapy as well as longitudinal differences between baseline and 3 months within the Responders group. Unclassified (unmapped) pathways were omitted. Non-parametric Cliff’s delta was calculated in R package *effectsize* to compile a list of pathways that display the greatest change/difference in each pairwise contrast. 95%CI was used to highlight significantly different pathways. MaAsLin2 was used as a complementary analysis to test therapy response-related effects and adjust results for age. Only pathways present in ≥10% of analyzed samples in abundance ≥0.01% were used for MaAsLin2, q-value threshold was set at 0.25 and p-value threshold at 0.05.

Sparse Partial Least Squares Discriminant Analysis (sPLS-DA) in *MixOmics* package^59^ (R, Bioconductor) was performed to identify functional pathways that explain the differences between the baseline and 3-month therapy within compliant individuals (Figure 4) as well as compliant vs non-compliant participants at each time-point (Figure 9). MixOmics output for Classification error rate (CER) was used to determine the number of components (i.e., clusters of pathways) best explaining the difference between the groups, followed by a balanced error rate (BER) estimate for the number of features (i.e., individual pathways) within each component. Cohen’s d parameter was then calculated for each pathway of the identified components using R package e*ffectsize* to visualize the differences in pathway coverage. Each pathway or the metabolite identified in sPLS-DA components was manually entered in Metacyc and PubChem public databases for pathway visualization and curation of the final figures.

### Metabolomics analysis in a patient subgroup

For a subgroup of 34 T2D patients we also performed targeted metabolomics in fecal samples at both time points. We measured: (1) SCFAs: acetic acid, propionic acid, isobutyric acid, butyric acid, isovaleric acid, valeric acid, and caproic acid; (2) bile acids: cholic acid (CA), chenodeoxycholic acid (CDCA), deoxycholic acid (DCA), glycocholic acid (GCA), glycochenodeoxycholic acid (GCDCA), glycodeoxycholic acid (GDCA), glycolithocholic acid (GLCA), lithocholic acid (LCA), taurocholic acid (TCA), taurochenodeoxycholic acid (TCDCA), taurodeoxycholic acid (TDCA), taurolithocholic acid (TLCA), and ursodeoxycholic acid (UDCA); (3) other measured metabolites include amino acids and other small molecules: acetylcarnitine, arginine, butyrylcarnitine, carnitine, carnosine, citrulline, creatinine, cystathionine, cystine, glutamic acid, glutamine, histidine, hydroxy-proline, isoleucine, isovalerylcarnitine, kynurenic acid, leucine, lysine, melatonin, methionine, myristoylcarnitine, octanoylcarnitine, ornithine, palmitoylcarnitine, phenylalanine, proline, propionylcarnitine, putrescine, serotonin, taurine, tryptophan, tyrosine, and valine.

### Sample processing for targeted metabolomics

For the targeted analysis of the Bile Acid panel and Amino Acid panel, 0.5 g of thawed fecal sample was mixed with 20 μl of ISTD AA mix (Metabolomics Amino Acid Mix Standard Cambridge Isotope Laboratories MSK-A2-1.2) and 1 ml of methanol. The sample was mixed thoroughly and centrifuged, 100 μl of supernatant was used for Bile Acid measurements, 400 μl of supernatant was subsequently mixed with 400 μl chloroform and 200 μl water, incubated on ice for 10 minutes and 500 μl of upper methanol phase was collected after centrifugation for Amino acid panel. For SCFAs measurements, 0,1 g of thawed fecal sample was mixed with 200 μl SCFA ITSD, 250 μl sulfuric acid 50% w/v, and 1 ml MTBE. After centrifugation, 500 μl of supernatant was used for measurements.

### Metabolomics instrumental analysis

#### SCFA measurements with GC-MS

The gas chromatography was conducted on an Agilent Technologies 6890 gas chromatograph (GC) equipped with a flame ionization detector (FID). Capillary column DB-FFAP (30 m × 0.25 mm × 0.25 µm) was used with helium as the carrier gas. The flow rate of carrier gas was 1.5 ml/min (at carrier gas pressure of 15–17 psi). The injector was operated at pulsed splitless mode (30 psi until 0.5 min with purge flow 200 ml/min for 1 min) using Agilent Technologies 7683B series injector with injection volume of 2 µl. The oven program was from 60°C (with 2 min hold) to 180°C at 15°C/min second ramp to 250°C at 50°C/min (with 3.6 min hold). The FID was maintained at 250°C and inlet at 275°C.

#### Amino acid measurements with UPHLC-MS

A Dionex 3000 HPLC system (Thermo Scientific) coupled with TSQ Quantis™ Triple Quadrupole (Thermo Scientific) mass spectrometer was used for the LC-MS/MS analysis. The chromatographic separation was carried out on an ACQUITY UPLC BEH Amide, 1.7 μm, 2.1 × 100 mm analytical column (Waters) equipped with a VanGuard: BEH C18, 2.1 × 5 mm pre-column (Waters). The column was maintained at a temperature of 40°C and the sample injection volume was 2 μl. The mobile phase consisted of phase A - 0.15% formic acid (v/v) in water, and phase B - 0.15% formic acid (v/v) in 85% acetonitrile (v/v) with 10 mM ammonium formate. The gradient elution with a flow rate of 0.4 ml/min was performed for a total analysis time of 17 min.

#### Bile acid measurements with UHPLC/MS/MS

A Dionex 3000 HPLC system (Thermo Scientific) coupled with TSQ Quantis™ Triple Quadrupole (Thermo Scientific) mass spectrometer was used for the LC-MS/MS analysis. The chromatographic separation was carried out on an ZORBAX Eclipse XDB, 1.8 μm, 2.1 × 50 mm analytical column (Agilent) equipped with a VanGuard: BEH C18, 2.1 × 5 mm pre-column (Waters). The column was maintained at a temperature of 40°C and the sample injection volume was 2 μl. The mobile phase consisted of phase A - 0.1% formic acid (v/v) in water, and phase B - 0.1% formic acid (v/v) in acetonitrile (v/v). The gradient elution with a flow rate of 0.4 ml/min was performed for a total analysis time of 10 min.

### Metabolomics data processing and statistical analysis

SCFA raw data processing was performed using MSD Chemstation D.03.00.611 (Agilent). Standard solutions of acetic acid, propionic acid, butyric acid, iso-butyric acid, pentanoic acid and iso-pentanoic acid (Acros) were prepared in 1–15000 µM concentrations for calibration curves. Calibration curves were expressed as the signal area ratio of analyte to internal standard and represented by a linear equation. The quality of automated peak integration and calibration was manually checked and adjusted where needed. The results were exported to Excel and used for further data analysis.

For amino acids and bile acids, the TraceFinder 4.1 software (Thermo Scientific) was used for data processing. A 7-point linear calibration curve with internal standardization and 1/x weighing was constructed for the quantification of the metabolites. The quality of automated peak integration and calibration was manually checked and adjusted where needed. The obtained metabolite concentration values in samples were reported as μM (μmol/L). The results were exported from TraceFinder 4.1 using the Excel report function and used for further data analysis.

Statistical analyses and visualizations were performed using MetaboAnalyst 6.0.^60^

For all quantified metabolite concentration (μM) data in each analysis for normalization we used Log transformation, Pareto scaling and in each contrast missing values were replaced by 1/5 of min positive values of their corresponding variables. We performed linear regression analysis for time series (before treatment, after 3-month long treatment) with two groups (Responders, Non-responders) design and controlling for age. The underlying method of linear regression in MetaboAnalyst 6.0 is based on limma, age was used as an adjusting covariate in the model matrix. Random Forest analysis (performed in MetaboAnalyst 6.0) was used to study non-linear relationships by using ensemble of classification trees and to calculate feature importance, which can be valuable for understanding the contribution of different variables to the classification. While constructing each tree, approximately one-third of the instances are excluded from the bootstrap sample. These excluded instances, known as out-of-bag (OOB) data, are subsequently utilized as a test sample to derive an impartial estimation of the classification error (OOB error). The assessment of variable importance involves determining the rise in the OOB error when the variable is permuted.

Additionally, we executed a Pathway and Biomarker analysis for metabolite measurements available at the MetaboAnalyst 6.0 platform. Pathway analysis in MetaboAnalyst 6 is based on MetPa tool,^61^ in all analyzed contrasts we applied the global test enrichment method and used relative-betweenness centrality for topology analysis, reference metabolome: Homo sapiens (KEGG pathway info was obtained in December 2023). For biomarker identification, we included the top 100 ranked (based on -p-values) computed metabolite ratios to improve biomarker discovery, since the ratios may carry more information than the two corresponding metabolite concentrations alone. We employed classical ROC analysis for univariate biomarker discovery, calculating AUC from ROC curves. MetaboAnalyst 6.0 was also used for supervised multivariate ROC analysis via Monte-Carlo cross-validation. This involves evaluating feature importance using two-thirds of the samples and building classification models with the top features, validated on the remaining one-third. Linear SVM classification and SVM feature ranking are utilized to find the optimal hyperplane for class separation. Features were selected recursively, with subsets decreasing in size over iterations, allowing evaluation of different combinations’ impact on classification. Feature importance was determined by the frequency of selection in the best classifier. FDR was calculated where applicable throughout the analyses performed for metabolomics data.

## Supplementary Material

Supplemental Material

## Data Availability

All shotgun metagenomic sequences are available from the European Nucleotide Archive (ENA) under the accession number PRJEB62817. Metabolomics data have been deposited under the accession link: https://rtucloud1-my.sharepoint.com/:f:/g/personal/orbitrap_rtu_lv/Eub98rgI0-1HsyxzH6VaBKEBhpkmfHmMWwTPbCos82b97w?e=ttlLgG..
