# Supplementary material for: Gut microbiome encoded purine and amino acid pathways present prospective biomarkers for predicting metformin therapy efficacy in newly diagnosed T2D patients
Source: Gut Microbes. 2024 Jun 13;16(1):2361491. doi: 10.1080/19490976.2024.2361491 (PMC11178274; doi:10.1080/19490976.2024.2361491)
Supplement: Supplemental Material [file KGMI_A_2361491_SM1991.zip › Supplementary/Supplementary file 8.docx]

Supplementary file 8. List of inclusion/exclusion criteria for the OPTIMED cohort

1. Inclusion criteria

- - - 1. Newly diagnosed type 2 diabetes mellitus and initiation of oral antidiabetic therapy;
      2. Previous diagnosis of type 2 diabetes mellitus and no oral antidiabetic or insulin therapy used in the previous three months;
      3. Newly diagnosed patients for glycemic control for an acute on-site intensive insulin therapy up to five days, continued afterwards;
      4. Patients unavailable and not optimized in drug trials;
      5. Age of 18;
      6. Patients meeting the diagnostic criteria for type 2 diabetes mellitus:
    1. Fasting blood glucose ≥7 mmol / l;
    2. Blood glucose two hours after OGTT with 75 g glucose ≥11.1 mmol / l.
       1. Prior to the study-related procedures, the consent of a person's participation in the clinical trial is received by submitting a signed and dated informed consent document.

2. Exclusion criteria:

1. Use of peroral antidiabetic therapy;
2. Use of Type 2 *diabetes mellitus* insulin therapy;
3. Pregnancy.

HbA_1c_ - hemoglobin A1c; OGTT – oral glucose tolerance test.
